# Special Analytical and Health Commission on Tinned and Preserved Food

**Published:** 1906-07-14

**Authors:** 


### SPECIAL ANALYTICAL AND HEALTH COMMISSION ON TINNED AND PRESERVED FOOD.

### III. CANNED FISH.

However important we may regard the proper preservation of meat, from the point of view of economics, the preservation of fish is certainly no less important. Fish, unlike cattle, want no pasture, and their abundance in certain places and at certain times of the year is proverbial; this variety of food is of all proteid or nitrogenous foods undoubtedly the most plentiful. Although from the chemical standpoint it contains less proteid nutriment weight for weight than butchers' meat, the cheap price at which it can be sold makes up for this. It may be reckoned, roughly, that 1½ lb. of ordinary white fish are about equivalent to one pound of meat. In making this calculation we must remember that in the case of fish, as ordinarily purchased, there is a good deal of waste material, skin, head, tail, scales, etc., this in some cases amounting to 70 per cent. This inedible residue differs widely in different kinds of fish, and hence some of the apparently dearest varieties of fish are often in reality the cheapest. Further, this consideration is not applicable to the matter actually before us, as in the case of canned or preserved fish the inedible residue is much reduced. Fish, and certainly canned fish, is an article of diet offering the greatest amount of nutriment for any given sum. If we turn from the chemistry of the subject to its physiology we find from that point of view that fish possesses certain advantages. It is blander than meat, containing less of the so-called extractives of stimulating purin bases; hence fish is generally chosen as a diet in cases in which you want to avoid stimulation of the spinal nervous centres, as it is also in those cases in which there is a tendency to the gouty diathesis, or to an accumulation in the body of substances of the purin type. Further, in the subjects of renal irritation or disease we chiefly prescribe fish instead of meat because these meat extractives tend to give the kidneys more work to do. Fish is also probably, weight for weight, more digestible than meat—that is, it requires less work on the part of the digestive organs to prepare it for absorption. Fish also contains a larger proportion of mineral matter than meat, and especially phosphates. To these phosphates originally much virtue was attached, and their relative excess in fish was held to endow this article of diet with special virtues as a brain food and as an aphrodisiac. This latter virtue was always attributed to it in the East, where they ought certainly to be judges. In the opinion of the writer, fish may be quite correctly regarded as a good nerve food, but the explanation of this probably lies in the fact of its organic phosphorus constituents, and is especially true of the ova of certain fish which are especially rich in glycerophosphates and lecithin. Among these we may cite caviar, the roe of the sturgeon, and ordinary herring or cods' roe. Fish is also relatively rich in fat. Eel is perhaps one of the fattest of fish, containing about 18 per cent. of fat, salmon coming next with 12 per cent., and a lean fish like whiting containing 2 per cent. The specific flavour of different fishes is mainly due to the presence of certain constituents of their fat. Fish fat is usually less rich in the solid compounds of the fatty acid series than animal fat.

The importance of the proper preservation of fish cannot be overestimated, as this food is most liable to undergo decomposition, and, moreover, the products of decomposition are in most cases deadly poisonous to the human system.

### The Samples Submitted.

We have received from Messrs. Travers and Sons, Limited, of 119 Cannon Street, E.C., who are the distributing agents in the United Kingdom for the Queensland Government, samples of canned sardines, salmon, and lobster. These goods are packed in British Columbia under Government inspection, and undergo a most severe test as regards quality. These fish are all preserved in cans, and we may say at once, before we describe the individual articles, that the samples which came into our hands were thoroughly well canned. The cans did not bulge and showed no blisters, percussion of their surface did not give a hyper-resonant note. The inside of the cans was bright, and the metal appeared to have remained entirely unattacked by the contents. In fact, before opening the tin there was no reason to suppose that its contents were not perfectly sound.

### Sardines.

The sardine is one of the most nutritious of fish. A good deal of our knowledge of the chemical composition of fish is due to Miss Catherine Williams, who analysed a number of fish cooked and uncooked. The sardine belongs to the class of fat fish; in it there is perhaps less waste than in any other fish; this is estimated at approximately only 5 per cent. König gives the following analytical results for the canned sardine from which the enveloping oil has been removed: Water, 53.64 per cent.; meat substances, 25.90 per cent.; fat, 11.27 per cent.; ash, 9.00 per cent. The sardines which were submitted to us (ship brand) were preserved in oil, air being by this means excluded. The sardines were of excellent taste and quality, and showed no sign of decomposition or coloration of any kind. We speak of coloration advisedly because preserved sardines occasionally become red, the colour being due to the development in large numbers of a chromogenic bacillus which is present occasionally on the skins of the sardines before preservation. This bacillus is, according to Auche, distinct from the well-known bacillus prodigiosus and is non-pathogenic.

### Canned Salmon.

The sample of canned salmon which we received from Messrs. Travers (ship brand) was of excellent quality. The salmon when fresh is an expensive fish. But it is not quite so dear as it seems, and the canned salmon is certainly one of the cheapest forms of nutriment extant. The waste in the salmon is only slightly more than that in the sardine, and is estimated at 6 per cent. The following is the composition of canned salmon (mean of three analyses): Water, 61.78 per cent.; meat substances, 20.16 per cent.; fat, 15.68 per cent.; ash, 2.38 per cent. Thus we see that the salmon is only slightly less nutritious, weight for weight, than the sardine. The salmon owes its flavour to a peculiar constituent of the fat, and its appetising colour to the fact that its muscle fibre contains, in addition to the hæmoglobin of its blood, a peculiar lipochrome which, according to Krukenberg and Wagner, is not a proteid substance.

### Canned Lobster.

We have received from Messrs. Travers a specimen (ship brand) of canned lobster. The lobster belongs to quite a different kind of fish to those just considered, and zoologically is more nearly allied to the oyster than to the salmon or sardine. It is a good example of the edible invertebrata. The flesh of those animals does not present any marked difference morphologically to that of other animals. Chemically, however, it contains rather more water, less fat, and more nitrogen free extractives. The following is an average composition of lobster flesh: Water, 81.84 per cent.; meat substances, 14.49 per cent.; fat, 1.49 per cent.; ash, 1.72 per cent.; nitrogen free extractives, 0.12 per cent. From these figures it is obvious that the lobster is less nutritious than the average fish, hence it is used dietetically more as an adjunct to than as a main constituent of diet. The flesh of those animals is rather coarser than that of the average fish, but the fibres are easily split up, and probably the lobster is not as indigestible as popular fancy thinks it. The use of vinegar with it certainly aids its digestion, and is hence dietetically justified. The horn of the lobster, which makes up the main part of the body as opposed to the claws and muscles, is rather poorer in proteid substances and richer in water than the former. We would conclude our remarks on this subject by saying that we regard canned fish as a most valuable food from the economic standpoint, and as containing a maximum value in nutritive return for the expenditure of a given sum of money.

### Bloater Creme.

We have already spoken in favourable terms of the sausages of Messrs. Traznier, Yallop, and Co., Limited. They have a bloater creme in stone pots which should suit the most fastidious. It is prepared from the best parts only of real Yarmouth bloaters, with a little butter and spice. The medical officer of health in Great Yarmouth has certified the premises of this firm as "suitable for the purpose of preparing wholesome human food—provided with impervious concrete flooring; the walls, tables, steamers, and all cooking apparatus were clean, and the sanitary arrangements for employees were suitable and in working order. The public may very well be reassured of the reliability of this firm's goods.

